# Percutaneous graft stent closure of a Profunda femoris mycotic aneurysm secondary to infective endocarditis: a case report

**DOI:** 10.1093/ehjcr/ytaf628

**Published:** 2025-12-02

**Authors:** Tezel Kovanci, Emrah Bayam, Anil Avci, Selahattin Akyol, Ramazan Kargin

**Affiliations:** Kartal Koşuyolu High Specialization Training and Research Hospital, Denizer Caddesi Cevizli Kavşağı No: 2, Cevizli/Kartal, Istanbul 34870; Kartal Koşuyolu High Specialization Training and Research Hospital, Denizer Caddesi Cevizli Kavşağı No: 2, Cevizli/Kartal, Istanbul 34870; Kartal Koşuyolu High Specialization Training and Research Hospital, Denizer Caddesi Cevizli Kavşağı No: 2, Cevizli/Kartal, Istanbul 34870; Kartal Koşuyolu High Specialization Training and Research Hospital, Denizer Caddesi Cevizli Kavşağı No: 2, Cevizli/Kartal, Istanbul 34870; Kartal Koşuyolu High Specialization Training and Research Hospital, Denizer Caddesi Cevizli Kavşağı No: 2, Cevizli/Kartal, Istanbul 34870

**Keywords:** Graft stent, Infective endocarditis, Mycotic aneurysm, Percutaneous closure, Case report

## Abstract

**Background:**

Mycotic aneurysms are rare complications of infective endocarditis, occurring in ∼2% of cases and often associated with high morbidity and mortality. Femoral, carotid, and iliac arteries are most commonly affected. When this complication arises, it further complicates the management and treatment of infective endocarditis, which is already a challenging condition to treat.

**Case summary:**

We report a 34-year-old male with a history of mechanical mitral valve replacement who presented with fever, fatigue, and loss of appetite. Due to a high clinical suspicion of infective endocarditis, empirical antibiotics were initiated accordingly. Blood cultures yielded *Pseudomonas aeruginosa*, and transoesophageal echocardiography revealed vegetation on the mitral prosthetic valve, confirming infective endocarditis. During hospitalization, the patient developed acute leg pain and a drop in haemoglobin levels. Computed tomography angiography revealed a large mycotic aneurysm of the right profunda femoris artery. Due to haemodynamic instability and suspected impending rupture, endovascular treatment was performed using a graft stent. Complete aneurysm exclusion was achieved without complications. The patient completed 8 weeks of intravenous antibiotics. Follow-up imaging confirmed resolution of vegetation, and no abscess or graft infection was observed in the femoral region. He was discharged in stable condition.

**Discussion:**

Mycotic aneurysms require urgent and aggressive management due to the risk of rupture. While surgical repair remains the standard of care, endovascular approaches such as graft stent placement offer a viable alternative in haemodynamically unstable patients or those with high surgical risk. This case highlights the importance of timely diagnosis, appropriate antibiotic therapy, and individualized interventional planning to achieve favourable outcomes.

Learning points

- The objective is to recognize mycotic aneurysm, a rare but life-threatening complication arising from infective endocarditis.
- Understanding the critical role of early diagnosis and appropriate antimicrobial therapy in the effective management of mycotic aneurysms.
- To learn which patient groups are appropriate candidates for surgical vs. percutaneous treatment options in the management of mycotic aneurysms.

## Introduction

Mycotic aneurysms are a rare complication of infective endocarditis. They occur in ∼2% of infective endocarditis cases and are associated with a poor prognosis and high mortality.^1^ Femoral, carotid, and iliac arteries are most commonly affected. *Staphylococcus aureus*, *Pseudomonas*, and *Salmonella* species are frequently implicated.^2^ These aneurysms also hinder effective infection control, as the aneurysmal lumen provides a favourable environment for bacterial proliferation. When this complication arises, it further complicates the management and treatment of infective endocarditis, which is already a challenging condition to treat.

## Summary figure

**Figure:**
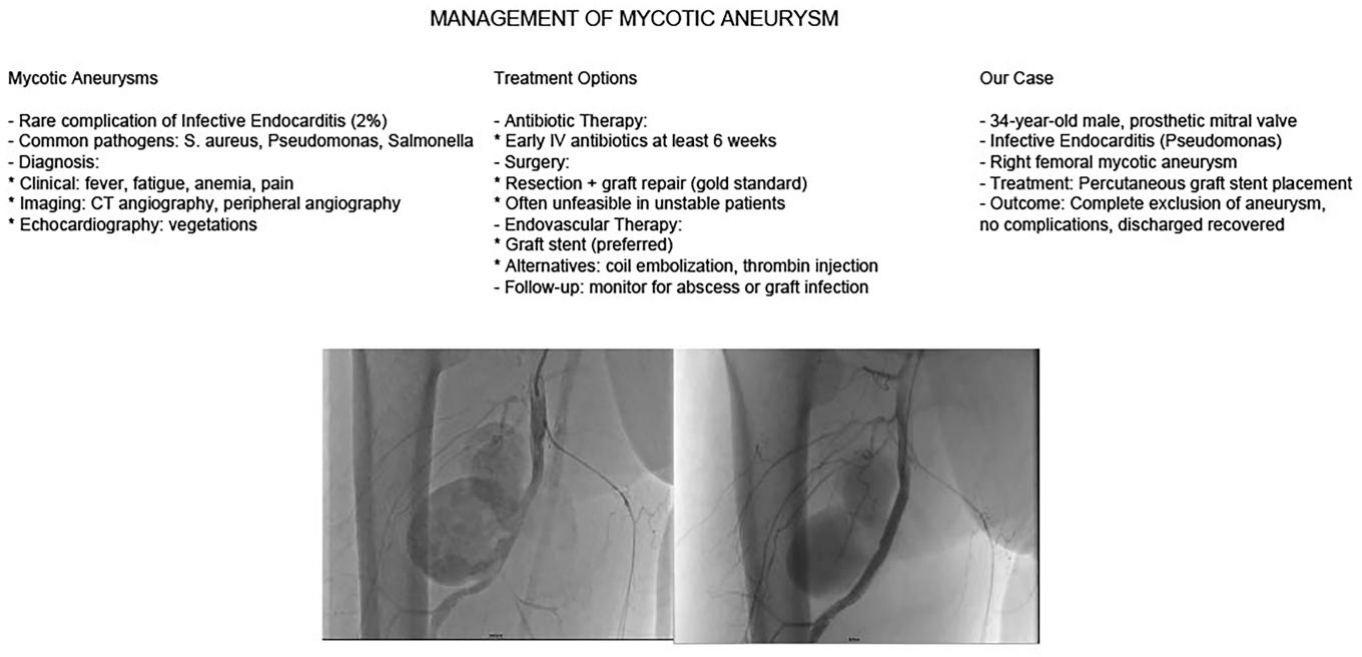


## Case presentation

**Figure 1:**
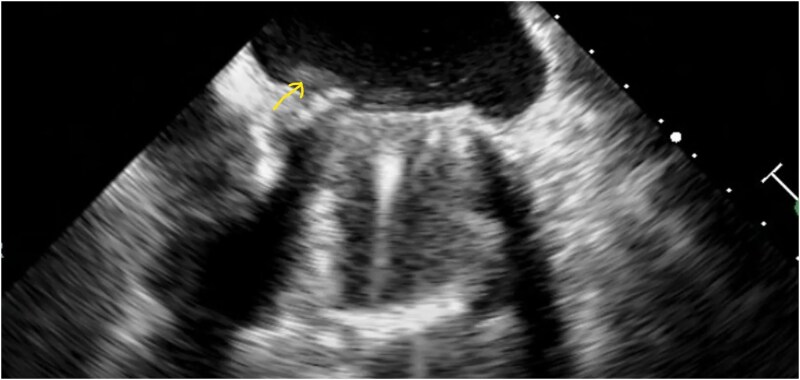
In the transoesophageal image, vegetation is observed on the mechanical mitral prosthetic valve.

**Figure 2:**
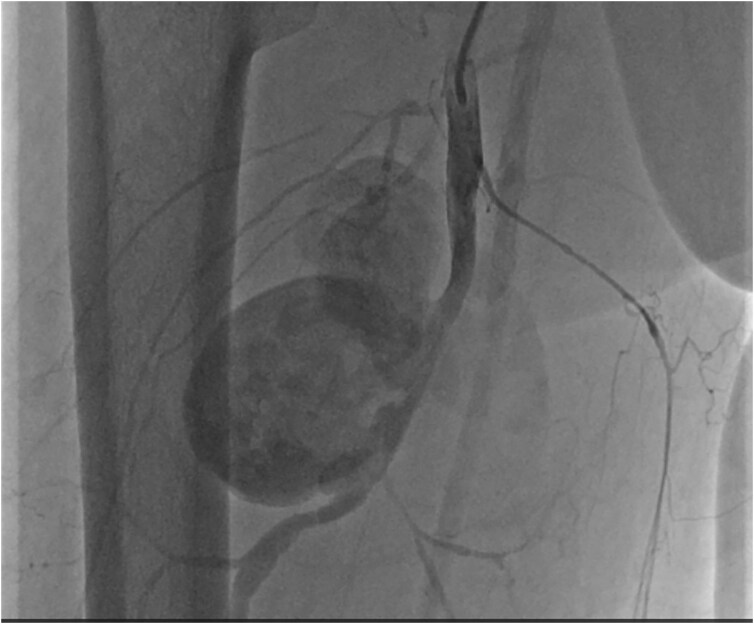
In peripheral angiographic imaging, a giant mycotic aneurysm was visualized in the right profunda femoris artery.

**Figure 3:**
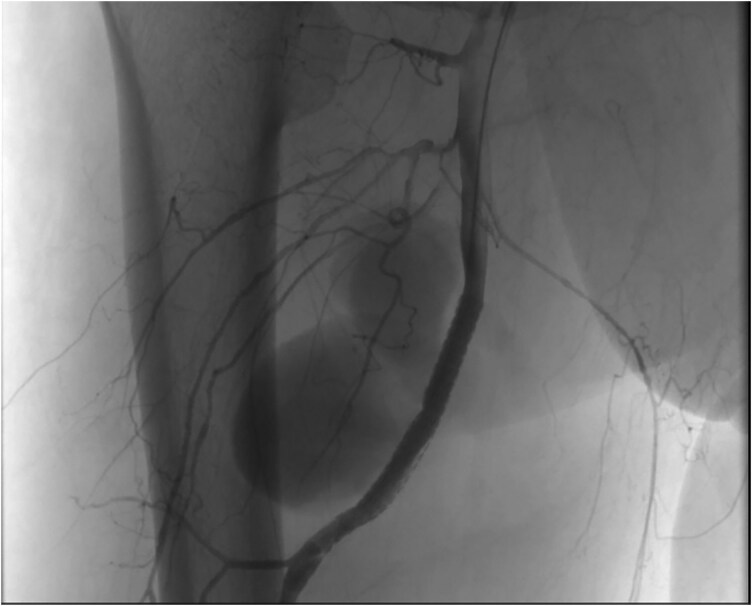
The profunda femoris artery was crossed with a 0.035-in. hydrophilic guidewire, and subsequently, a 6.0 × 60 mm graft stent was positioned to fully cover the ostium of the aneurysm. After stent deployment, exclusion of the aneurysm was observed, and no contrast extravasation was detected.

A 34-year-old male patient presented to our emergency department with complaints of fever (39°C), fatigue, and loss of appetite for the past 2 days. The patient had a history of mechanical mitral valve replacement 3 years ago, which was indicated for severe mitral stenosis resulting from rheumatic valvular disease. Initial investigations revealed that the patient’s electrocardiogram (ECG) was in normal sinus rhythm. Laboratory tests were unremarkable. However, C-reactive protein levels were found to be elevated. Transthoracic echocardiography demonstrated findings similar to previous imaging after the surgery. Due to a clinical suspicion of infective endocarditis, the patient was admitted to the cardiology department for further evaluation. Blood cultures were obtained, and empirical antibiotic therapy with intravenous vancomycin 500 mg twice a day and gentamicin 240 mg once a day was initiated. On follow-up after 1 week, *Pseudomonas aeruginosa* was isolated in the blood culture. Following consultation with the infectious diseases team, the antibiotic regimen was adjusted according to sensitivity testing, continuing with intravenous gentamicin 240 mg once a day. Transoesophageal echocardiographic evaluation revealed a mass measuring 1.2 × 1.4 cm consistent with vegetation on the mitral mechanical valve (*Figure 1*, *Video 1a* and *b*). Thus, the diagnosis of infective endocarditis was confirmed. On the 15th day of treatment, blood cultures remained negative. In clinical follow-up, the patient complained of leg pain, and a decrease in haemoglobin levels was noted in follow-up laboratory tests. Consequently, contrast-enhanced computed tomography angiography was performed and demonstrated a mycotic aneurysm in the right profunda femoris artery while excluding additional mycotic aneurysms or abscesses in other organ systems. The cardiovascular surgery department was consulted for possible surgical intervention; however, surgery was not performed due to ongoing haemoglobin decline, suspicion of rupture, and haemodynamic instability secondary to bleeding. Subsequently, the patient was taken to the catheterization laboratory, where peripheral angiography was performed. The angiographic evaluation revealed a large aneurysm originating from the profunda femoris artery (*Figure 2*). Although active extravasation was not observed on imaging, the team decided to proceed with stent exclusion due to the large size of the aneurysm, ongoing haemoglobin decline, and high risk of rupture. A hydrophilic 0.035-in. guidewire was advanced into the profunda femoris artery, and a graft stent was deployed to cover the neck of the aneurysm. Post-implantation control angiography demonstrated complete exclusion of the aneurysm, and the procedure was completed (*Figure 3*). During follow-up, the patient’s haemodynamic status improved. Intravenous gentamicin therapy was completed over 8 weeks. Repeated blood cultures remained negative. Follow-up transoesophageal echocardiography revealed complete resolution of the previously detected vegetation on the mechanical mitral valve. Following the completion of antibiotic therapy, the patient was discharged in complete recovery, and evaluation at the 4-month outpatient follow-up confirmed a favourable clinical course, with no evidence of complications, abscess formation, or recurrence in the femoral region.

## Discussion

The term *mycotic* was first introduced by William Osler in 1885 to describe a form of endarteritis associated with infection of the aneurysmal arterial wall. The aetiopathogenesis involves the invasion of microorganisms into the intimal layer, initiating an inflammatory cascade that progressively extends through the media and adventitia. This process results in structural weakening and destruction of the vessel wall, predisposing it to aneurysmal dilatation and potential rupture. A number of clinical conditions, including immunosuppression, human immunodeficiency virus infection, infective endocarditis, and septic arthritis, have been recognized as important predisposing factors in the development of mycotic aneurysms. *Staphylococcus aureus*, *Pseudomonas*, and *Salmonella* species are frequently implicated. Mycotic aneurysms are rare but fatal complications of infective endocarditis, occurring in ∼2% of cases. They hinder effective infection control, as blood stasis within the aneurysmal lumen provides a favourable environment for bacterial proliferation and studies have shown that nearly half of mycotic aneurysms secondary to endocarditis rupture either early or late during follow-up. Aneurysmal rupture is associated with a poorer prognosis and more severe clinical outcomes. Therefore, they should be treated urgently without delay, as delayed intervention significantly increases the risk of rupture and worsens clinical outcomes. The management of mycotic aneurysms remains challenging. According to current literature, the treatment of mycotic aneurysms typically involves prolonged antibiotic therapy in combination with interventional procedures. Prompt initiation of broad-spectrum antibiotic therapy significantly reduces all endocarditis-related complications, including the risk of developing mycotic aneurysms and subsequent rupture.^3^ Definitive therapy traditionally involves surgical resection with debridement and vascular reconstruction. However, high surgical risk, ongoing sepsis, and haemodynamic instability in cases of rupture may render surgical intervention unfeasible for some patients.^4^ There are only a limited number of case reports in the literature describing closure of mycotic aneurysm using percutaneous techniques such as coil embolization, thrombin injection, and graft stent placement. Compared to coil embolization and thrombin injection, graft stent placement is generally considered both more technically feasible and more effective in achieving successful aneurysm exclusion. The main concern in such procedures is the potential for the occluded infected lumen to evolve into an abscess or graft infection. To prevent such complications, percutaneous interventions should be accompanied by prolonged antibiotic therapy. A minimum of 6 weeks of intravenous antibiotic treatment is recommended for infective endocarditis-associated mycotic aneurysms. In high-risk or immunocompromised patients, prophylactic antibiotic therapy may be extended up to 6 months to reduce the risk of relapse or graft-related infections.^5–7^ As in our case, the patient was haemodynamically unstable and not a candidate for immediate surgery. Therefore, a graft stent was successfully placed as a bailout treatment. Notably, the intervention was performed after blood cultures had turned negative and under ongoing antibiotic therapy, which likely contributed to the favourable outcome. In cases requiring urgent intervention where surgical risk is high and the patient is haemodynamically unstable due to rupture, percutaneous closure techniques particularly graft stent replacement should be considered as a therapeutic option for bailout management with a combination of prolonged antibiotic therapy.

## Conclusion

Mycotic aneurysms are rare complications of infective endocarditis and associated with a poor prognosis and high mortality. Due to the risk of rupture, they require urgent and aggressive management. Early diagnosis and prompt initiation of antibiotic therapy play a crucial role in clinical management. Surgical intervention remains the standard approach in the treatment of mycotic aneurysms, but it is not always feasible due to high surgical risk, haemodynamic instability, or rupture. Percutaneous techniques, particularly graft stent placement, offer an alternative therapy in this patient population.

## Supplementary Material

ytaf628_Supplementary_Data

## Data Availability

The data supporting the findings of this case report are available and can be shared upon reasonable request from the corresponding author. All data have been anonymized to protect patient confidentiality.

## Lead author biography

Dr Tezel Kovanci, born on June 26, 1997, in the Turkish Republic of Northern Cyprus, completed his secondary education in Cyprus. He earned his medical degree from Çukurova University Faculty of Medicine in 2021. Since then, he has been undergoing residency training in cardiology at Koşuyolu Heart Hospital in Istanbul (2021–25).

## Supplementary material

**Consent:** The authors confirm that written consent for submission and publication of this case report including images and associated text has been obtained from the patient, in line with the COPE guidelines.

**Funding:** The authors declare that this study received no financial support.
